# Analysis of Maternal Prenatal Weight and Offspring Cognition and Behavior: Results From the Promotion of Breastfeeding Intervention Trial (PROBIT) Cohort

**DOI:** 10.1001/jamanetworkopen.2021.21429

**Published:** 2021-08-19

**Authors:** Emily Oken, Jennifer W. Thompson, Sheryl L. Rifas-Shiman, Konstantin Vilchuk, Natalia Bogdanovich, Mikhail Hameza, Seungmi Yang, Rita Patel, Michael S. Kramer, Richard M. Martin

**Affiliations:** 1 Division of Chronic Disease Research Across the Lifecourse, Department of Population Medicine, Harvard Medical School, Harvard Pilgrim Health Care Institute, Boston, Massachusetts; 2 The National Research and Applied Medicine Mother and Child Centre, Minsk, Belarus; 3 Department of Epidemiology, Biostatistics and Occupational Health, Faculty of Medicine, McGill University, Montreal, Quebec, Canada; 4 Musculoskeletal Research Unit, Translational Health Sciences, Bristol Medical School, University of Bristol, Bristol, United Kingdom; 5 Department of Pediatrics, Faculty of Medicine, McGill University, Montreal, Quebec, Canada; 6 Department of Population Health Sciences, Bristol Medical School, University of Bristol, Bristol, United Kingdom; 7 National Institute for Health Research Biomedical Research Centre, University Hospitals Bristol and Weston National Health Service Foundation Trust, University of Bristol, Bristol, United Kingdom; 8 Medical Research Council Integrative Epidemiology Unit, University of Bristol, Bristol, United Kingdom

## Abstract

**Importance:**

Prenatal experiences can influence fetal brain development.

**Objective:**

To examine associations of maternal prenatal body mass index (BMI) with cognition and behavior of offspring born full-term.

**Design, Setting, and Participants:**

This cohort study examined follow-up data from a breastfeeding promotion intervention at 31 hospitals and affiliated polyclinics in the Republic of Belarus. Participants included 11 276 children who were evaluated from birth (1996-1997) to adolescence (2017-2019), with maternal BMI information available in prenatal medical records.

**Exposures:**

Maternal BMI, calculated as weight in kilograms divided by height in meters squared, after 35 weeks gestation; secondary analyses examined maternal BMI at other time points and paternal BMI.

**Main Outcomes and Measures:**

Trained pediatricians assessed child cognition with the Wechsler Abbreviated Scales of Intelligence (WASI) at 6.5 years and the computerized self-administered NeuroTrax battery at 16 years, both with an approximate mean (SD) of 100 (15). Parents and teachers rated behaviors at 6.5 years using the Strengths and Difficulties Questionnaire (SDQ, range 0-40). Mixed-effects linear regression analyses corrected for clustering, adjusted for the randomized intervention group and baseline parental sociodemographic characteristics, and were considered mediation by child BMI.

**Results:**

Among 11 276 participants, 9355 women (83%) were aged 20 to 34 years, 10 128 (89.8%) were married, and 11 050 (98.0%) did not smoke during pregnancy. Each 5-unit increase in of maternal late-pregnancy BMI (mean [SD], 27.2 [3.8]) was associated with lower offspring WASI performance intelligence quotient (IQ) (−0.52 points; 95% CI, −0.87 to −0.17 points) at 6.5 years and lower scores on 5 of 7 NeuroTrax subscales and the global cognitive score at 16 years (−0.67 points; 95% CI, −1.06 to −0.29 points). Results were similar after adjustment for sociodemographic characteristics, pregnancy complications, and paternal BMI and were not mediated by child weight. Higher late pregnancy maternal BMI was also associated with more behavioral problems reported on the SDQ by teachers but not associated with parent-reported behaviors (externalizing behaviors: 0.13 points; 95% CI, 0.02 to 0.24 points; and total difficulties: 0.14 points, 95% CI, −0.02 to 0.30 points). Results were similar for maternal BMI measured in the first trimester or postpartum. In contrast, higher 6.5-year paternal BMI was associated with slightly better child cognition (WASI verbal IQ: 0.42 points; 95% CI, 0.02 to 0.82 points; NeuroTrax executive function score: 0.68 points; 95% CI, 0.24 to 1.12 points) and fewer teacher-reported behavioral problems (total difficulties: −0.29 points; 95% CI, −0.46 to −0.11 points).

**Conclusions and Relevance:**

This cohort study supports findings from animal experiments and human observational studies in settings with higher maternal BMI and obesity rates. Higher maternal prenatal BMI may be associated with poorer offspring brain development, although residual confounding cannot be excluded.

## Key Points

**Question:**

Is maternal prenatal weight associated with offspring cognitive ability and behavior?

**Findings:**

In this cohort study of 11 276 Belarusian children, higher maternal body mass index after 35 weeks’ gestation was associated with a slightly lower child intelligence quotient at 6.5 years and lower cognitive scores in multiple domains at 16 years. Associations were not mediated by child weight and were robust to adjustment for sociodemographic characteristics, pregnancy complications, and paternal weight.

**Meaning:**

These findings suggest that higher maternal body mass index during pregnancy may adversely influence offspring brain development.

## Teaser

This cohort study examines association of maternal prenatal body mass index with cognition and behavior of offspring born full-term.

## Introduction

A range of early childhood exposures has been associated with cognition and behavior, including environmental toxicants,^1^ psychosocial stress,^2^ and infant nutrition.^3^ A fetus may be even more sensitive to environmental insults.^4,5,6,7^ Given that the global prevalence of obesity among women increased from 6% in 1975 to 15% in 2016,^8^ an area of ongoing interest is the extent to which higher maternal weight status in pregnancy might influence the developing brain.

Animal experiments demonstrate the effects of maternal high-fat diets or obesity during pregnancy on abnormal offspring behavior.^5^ Similarly, a 2018 systematic review of human observational studies found that the odds for any adverse neurodevelopmental outcome was 17% higher (95% CI, 1.11-1.24) among children of mothers with overweight and 51% higher (95% CI, 1.35-1.69) among children born to mothers with obesity before pregnancy relative to children born to mothers with normal weight.^9^ However, outcomes were dichotomized, and most were assessed at relatively young ages. Additionally, many studies used retrospective self-reports of maternal weight or maternal reports of offspring outcomes, which are subject to measurement error and could introduce bias. Finally, all studies were set in high-income countries (ie, US, Western Europe, Australia) with high obesity prevalence.^8^

We examined associations of maternal prenatal weight status with offspring cognitive ability and behavior in a setting with low overall prevalence of both maternal and child obesity.^10,11,12^ We leveraged data from a large longitudinal birth cohort nested within a randomized trial, with recorded maternal prenatal weight, high rates of follow-up, and standardized research measures of cognition and behavior in childhood and adolescence. We hypothesized that higher maternal prenatal body mass index (BMI), calculated as weight in kilograms divided by height in meters squared, would be associated with poorer cognitive and behavioral outcomes.

## Methods

This is a cohort study of longitudinal data from the Promotion of Breastfeeding Intervention Trial (PROBIT), a cluster-randomized controlled trial (ISRCTN37687716) of a breastfeeding promotion intervention in the Republic of Belarus from 1996 to 1997.^3,13^ PROBIT was approved by the Belarusian Ministry of Health, the McGill University Health Centre research ethics board, the institutional review board at Harvard Pilgrim Health Care, and the Avon Longitudinal Study of Parents and Children Law and Ethics Committee at the University of Bristol. A parent or legal guardian provided written informed consent in Russian at enrollment and at each follow-up visit, and participants provided written assent at 11.5 years and 16 years. This report follows the Strengthening the Reporting of Observational Studies in Epidemiology (STROBE) reporting guideline.

### Study Design and Participants

 Mothers were eligible for participation if they initiated breastfeeding, had no illnesses that would contraindicate or severely compromise breastfeeding, had a healthy singleton infant of 37 weeks’ or more gestation, 2500 g or more birth weight, and Apgar score 5 or higher at 5 minutes. An estimated 1% to 2% of women who were eligible declined participation. All study data were collected at participating maternity hospitals and polyclinics by Belarusian physicians who participated in detailed in-person training with periodic refreshers in Minsk, the Belarusian capital. Monitoring visits and data audits ensured data reliability.^3,10,13,14,15^

### Maternal Body Mass Index

Study physicians abstracted maternal weights and heights from clinical prenatal records. We calculated maternal late-pregnancy BMI using the highest weight recorded after 35 weeks’ gestation. We used maternal height measured at a research visit at 11.5 years postpartum; if it was missing, we substituted height recorded in the mother’s prenatal record. We also calculated first trimester BMI using the earliest recorded weight, which was available for 2346 mothers.

At the 6.5-year study visits, pediatricians asked mothers to self-report their current height and weight. At the 11.5-year visits, the pediatricians in this study measured the height and weight of the accompanying mother (Tanita TBF 300GS scale and Medtechnika stadiometer).^11^

### Cognitive and Behavioral Outcome Assessments

At the 6.5-year visit (range, 5.6-8.5 years), pediatricians assessed cognitive development using the Wechsler Abbreviated Scales of Intelligence (WASI).^14,16^ The WASI consists of 4 subtests (ie, vocabulary, similarities, block designs, and matrices), each reported on a conventional intelligence quotient (IQ) scale, with a population mean (SD) of 100 (15). We also administered the Strengths and Difficulties Questionnaire (SDQ), a validated screening tool designed to detect behavioral strengths and difficulties of children ages 4 to 16 years.^17,18^ The SDQ evaluates child behavior over the previous 6 months and consists of 5 subscales: emotional symptoms, conduct problems, hyperactivity/ inattention, peer problems, and prosocial behavior. Parents and teachers of the participants were both asked to complete the SDQ questionnaire. As in previous analyses,^19^ we combined the scores for hyperactivity and conduct problems to create an externalizing behavior problem score, and we combined the scores for emotional and peer problems to create an internalizing behavior problems score, with a possible range of 0 to 20 for each. These have been found to identify discrete behavioral domains.^17^ Total difficulties scores were created by summing the externalizing and internalizing problematic behavior scores, excluding prosocial behaviors, with a possible range of 0 to 40 for total difficulties scores, where higher scores denote more behavioral problems. For the prosocial scale (ie, range, 0-10), higher scores indicate better behaviors. The SDQ has high internal consistency, with a mean Cronbach α = .73 in a validation study,^17^ α = .74 in PROBIT for the parent report, and α = .86 for teacher report of total difficulties.

At the 16-year follow-up visit (2017-2019), neurocognitive function was assessed using a self-administered computerized battery of the NeuroTrax tests (NeuroTrax Corp).^20^ The NeuroTrax test consists of 10 short subtests that assess immediate and delayed verbal and nonverbal memory, word recognition, executive function, visual-spatial orientation, information-processing speed, and fine motor skills, which can be summarized into a global cognitive score. Age-standardized neurocognitive ability scores were computed from raw data using automatic algorithms and scaled to an IQ-type score.

### School Standardized Examination Results

Pediatricians requested the participants’ results on their national standardized examinations, which students in Belarus take between ages 15 to 16 years after 9 years of school or, if they remain in school, at ages 17 to 18 years after 11 years of school. Each result is reported as a single overall score (ie, 0-10 points) with higher scores denoting better examination results.

### Other Variables

At enrollment, study physicians collected information from hospital records and mothers, including infant sex; delivery date and mode; gestational diabetes or pregnancy-induced hypertension; birth weight and length; number of older siblings; type of infant feeding; maternal smoking during pregnancy and marital status; and the parents’ education, occupation, and marital status. We measured each child’s weight and height at all outcome visits and calculated their BMI.^21^

### Statistical Analyses

We used mixed-effects linear regression models to assess associations between maternal BMI and offspring cognitive and behavioral outcomes. The primary exposure for these analyses was late-pregnancy BMI, using the highest weight recorded after 35 weeks of gestation and before delivery. We first analyzed late-pregnancy BMI in quartiles to establish whether the shape of the association appeared linear. After establishing that associations of outcomes with maternal BMI, when they existed, were roughly linear across quartiles, we analyzed BMI exposures as continuous variables. For all analyses, we used mixed-effects models that included a random effect to account for the clustering of outcomes within polyclinics. In our primary analysis, we further adjusted for randomization to intervention or control groups, as well as for the following baseline variables hypothesized to be confounders: maternal age, height and smoking; maternal and paternal education and occupation; the number of older siblings; participant sex; and geographic location of residence. Adjustment for gestational age at late-pregnancy BMI did not affect estimates so we did not include this variable. No baseline covariates had missing data. We accounted for missing data on pregnancy complications, paternal BMI, and child BMI using multiple imputation with 50 iterations. We report unstandardized effect estimates with CIs to estimate magnitudes of associations and did not adjust for multiple comparisons given the correlated outcomes.

In separate sensitivity analyses, we further adjusted for gestational age at late-pregnancy BMI, paternal BMI based on height and weight reported by mothers at 6.5 years, and for pregnancy complications recorded in the prenatal medical record or self-reported on postpartum questionnaires (gestational diabetes and pregnancy-induced hypertension). To assess possible mediation by the child’s weight, we adjusted for child BMI at the time of the cognitive assessment. We also examined associations with maternal BMI assessed at different time points (ie, first trimester, 6.5 years, and 11.5 years), and with paternal BMI reported at 6.5 years, both unadjusted and adjusted for maternal late-pregnancy BMI.

We additionally performed an intention-to-treat analysis examining the effect of the randomized breastfeeding intervention on the school standardized examination score results, which has not previously been reported. We conducted all data analyses using SAS version 9.4 (SAS Institute, Inc).

## Results

Among 11 276 participants, 9355 women (83%) were aged 20 to 34 years, 10 128 (89.8%) were married, and 11 050 (98.0%) did not smoke during pregnancy. Mothers in the highest vs lowest quartile of late-pregnancy BMI were more likely to be 35 years or older (229 [8.1%] vs 35 [1.3%]) and less likely to be nulliparous vs having previously given birth (1187 [41.8%] vs 1945 [69.3%]), to smoke during pregnancy vs not smoking during pregnancy (49 [1.7%] vs 75 [2.7%]), or have a girl vs have a boy (1346 [47.4%] vs 1391 [49.6%]). No trends in breastfeeding duration or exclusivity were evident across maternal BMI quartiles (Table 1). There were slight differences in characteristics of participants included vs excluded from this analysis (eTable 1 in the Supplement).

**Table 1.** Characteristics of 11 276 PROBIT Participants Overall and According to Quartile of Maternal Late-Pregnancy BMI

Characteristics	No. (%)				
	All (n = 11 276)	Maternal late-pregnancy BMI quartile			
		1 (n = 2806)	2 (n = 2822)	3 (n = 2809)	4 (n = 2839)
**Maternal**					
Age at delivery, y					
<20	1471 (13.1)	545 (19.4)	403 (14.3)	327 (11.6)	196 (6.9)
20-34	9355 (83.0)	2226 (79.3)	2355 (83.5)	2360 (84.0)	2414 (85.0)
≥35	450 (4.0)	35 (1.3)	64 (2.3)	122 (4.3)	229 (8.1)
Education					
University					
Completed	1506 (13.4)	317 (11.3)	397 (14.1)	389 (13.9)	403 (14.2)
Incomplete	5827 (51.7)	1444 (51.5)	1429 (50.6)	1464 (52.1)	1490 (52.5)
Secondary					
Completed	3570 (31.7)	913 (32.5)	891 (31.6)	880 (31.3)	886 (31.2)
Incomplete	373 (3.3)	132 (4.7)	105 (3.7)	76 (2.7)	60 (2.1)
Occupation					
Nonmanual	4923 (43.7)	1154 (41.1)	1221 (43.3)	1238 (44.1)	1310 (46.1)
Manual	3817 (33.9)	828 (29.5)	937 (33.2)	983 (36.0)	1069 (37.7)
Student	321 (2.9)	126 (4.5)	94 (3.3)	68 (2.4)	33 (1.2)
Unemployed	2215 (19.6)	698 (24.9)	570 (20.2)	520 (18.5)	427 (15.0)
Marital status					
Married	10128 (89.8)	2480 (88.4)	2556 (90.6)	2511 (89.4)	2581 (90.9)
Cohabitating	725 (6.4)	205 (7.3)	165 (5.9)	182 (6.5)	173 (6.1)
Not married	423 (3.8)	121 (4.3)	101 (3.6)	116 (4.1)	85 (3.0)
Smoked during pregnancy	226 (2.0)	75 (2.7)	51 (1.8)	51 (1.8)	49 (1.7)
Gestational diabetes	441 (3.9)	103 (3.7)	95 (3.4)	111 (4.0)	132 (4.7)
Pregnancy-induced hypertension	371 (3.3)	31 (1.1)	61 (2.2)	91 (3.2)	188 (6.6)
BMI, mean (SD)					
First trimester^a^	23.0 (3.9)	19.5 (1.7)	21.4 (1.5)	23.2 (1.6)	27.8 (3.7)
Late pregnancy	27.2 (3.8)	23.1 (1.3)	25.6 (0.6)	27.8 (0.7)	32.2 (2.9)
Gestational age at late-pregnancy BMI, mean (SD), wk	39.0 (1.2)	38.9 (1.1)	39.0 (1.1)	39.0 (1.2)	39.0 (1.2)
Self-reported BMI at 6.5 y visit, mean (SD)^a^	24.4 (4.3)	21.3 (2.7)	22.9 (2.8)	24.6 (3.1)	28.6 (4.5)
BMI measured at 11.5 y visit, mean (SD)^a^	26.7 (5.7)	22.6 (3.3)	24.7 (3.5)	27.1 (4.1)	32.3 (5.9)
Height, mean (SD), cm^b^	163.9 (5.8)	165.0 (6.0)	164.0 (5.7)	163.5 (5.8)	163.2 (5.7)
BMI >30					
At 6.5 y visit	1159 (11.1)	17 (0.7)	48 (1.8)	148 (5.7)	946 (35.5)
At 11.5 y visit	1533 (24.0)	54 (3.3)	128 (8.1)	335 (21.3)	1016 (62.9)
**Paternal**					
Education					
University					
Completed	1381 (12.3)	347 (12.4)	373 (13.2)	360 (12.8)	301 (10.6)
Incomplete	283 (2.5)	96 (3.4)	82 (2.9)	59 (2.1)	46 (1.6)
Secondary					
Completed	9029 (80.1)	2195 (78.2)	2233 (79.1)	2231 (79.4)	2370 (83.5)
Incomplete	224 (2.0)	70 (2.5)	50 (1.8)	55 (2.0)	49 (1.7)
Missing	359 (3.2)	98 (3.5)	84 (3.0	104 (3.7)	73 (2.6)
Occupation					
Nonmanual	3161 (28.0)	817 (29.1)	822 (29.1)	783 (27.9)	739 (26.0)
Manual	6098 (54.1)	1404 (50.0)	1497 (53.1)	1544 (55.0)	1653 (58.2)
Student	138 (1.2)	50 (1.8)	44 (1.6)	23 (0.8)	21 (0.7)
Unemployed	1431 (12.7)	413 (14.7)	353 (12.5)	330 (11.8)	335 (11.8)
Unknown	448 (4.0)	122 (4.4)	106 (3.8)	129 (4.6)	91 (3.2)
Maternal-reported BMI at 6.5 y visit, mean (SD)	25.7 (3.3)	25.2 (3.1)	25.6 (3.3)	25.7 (3.2)	26.3 (3.5)
BMI >30 at 6.5 y visit	922 (9.7)	162 (7.0)	226 (9.5)	228 (9.6)	306 (12.4)
**Child**					
Assigned to intervention	6243 (55.4)	1561 (55.6)	1594 (56.5)	1525 (54.3)	1563 (55.1)
Female	5421 (48.1)	1391 (49.6)	1356 (48.0)	1328 (47.3)	1346 (47.4)
Gestational age, wk					
37-38	2035 (18.1)	598 (21.3)	502 (17.8)	478 (17.0)	457 (16.1)
39-41	8314 (73.7)	2022 (72.1)	2088 (74.0)	2097 (74.7)	2107 (74.2)
42-43	927 (8.2)	186 (6.6)	232 (8.2)	234 (8.3)	275 (9.7)
Birth weight, mean (SD), g	3444 (415.9)	3294 (376.3)	3415 (394.3)	3488 (399.5)	3578 (438.0)
Older siblings					
0	6409 (56.8)	1945 (69.3)	1738 (61.6)	1539 (54.8)	1187 (41.8)
1	3960 (35.1)	740 (26.4)	909 (32.2)	1029 (36.6)	1282 (45.2)
≥2	907 (8.1)	121 (4.3)	175 (6.2)	241 (8.6)	370 (13.0)
Location of residence					
East					
Urban	3646 (32.3)	975 (34.8)	936 (33.2)	872 (31.0)	863 (30.4)
Rural	1446 (12.8)	333 (11.9)	375 (13.3)	377 (13.4)	361 (12.7)
West					
Urban	2892 (25.7)	711 (25.3)	724 (25.6)	723 (25.7)	734 (25.9)
Rural	3292 (29.2)	797 (28.1)	787 (27.9)	837 (29.8)	881 (31.0)
Duration of any breastfeeding, mo					
<3	3810 (34.0)	960 (34.4)	927 (33.0)	953 (34.1)	970 (34.3)
3 to <6	2607 (23.2)	696 (24.9)	654 (23.3)	628 (22.5)	629 (22.3)
≥6	4800 (42.8)	1137 (40.7)	1225 (43.7)	1212 (43.4)	1226 (43.4)
Duration of exclusive breastfeeding, mo					
<3	8015 (71.5)	2042 (73.1)	1948 (69.4)	2003 (71.7)	2022 (71.6)
≥3	3201 (28.5)	750 (26.9)	858 (30.6)	791 (28.3)	802 (28.4)

Abbreviation: BMI, body mass index (calculated as weight in kilograms divided by height in meters squared).

^a^ N = 2346 for maternal first trimester BMI, 10 455 for 6.5-year BMI, and 6398 for 11.5-year BMI.

^b^ Height measured at 11.5-year postpartum research visit or recorded in prenatal record if research height not available.

Mean (SD) first trimester BMI among 2346 mothers was 23.0 (3.9) and BMI after 35 weeks’ gestation was 27.2 (3.8). Maternal obesity (ie, BMI ≥ 30) prevalence was 1159 (11.1%) at 6.5 years based on self-report and 1533 (24.0%) at 11.5 years based on research measures. All maternal BMI measures were strongly intercorrelated (Table 1; eTable 2 in the Supplement). Maternal late-pregnancy BMI was also correlated with child BMI measures (Pearson *r* range, 0.22-0.24) but less so with paternal BMI (Pearson *r* = 0.13).

Associations of maternal late-pregnancy BMI with sociodemographic markers were mixed. The heaviest quartile vs lowest quartile was more likely to have completed university (403 [14.2%] vs 317 [11.3%]) and less likely to be unemployed (427 [15.0%] vs 698 [24.9%]) or unmarried (85 [3.0%] vs 121 [4.3%]), suggesting higher sociodemographic status. However, mothers in the heaviest quartile were also more likely to have a manual occupation (1069 [37.7%] vs 828 [29.5%]) and had partners who were less likely to have completed university (301 [10.6%] vs 347 [12.4%]) (Table 1). Higher maternal BMI at 6.5 years was associated with lower socioeconomic measures, whereas higher paternal and child BMI were associated with higher socioeconomic measures (eFigure in the Supplement), as previously reported.^12^

At 6.5 years, the mean (SD) full-scale WASI was 106.6 (16.0) points, the mean (SD) parent-rated total SDQ score was 11.5 (5.0) points, and the mean (SD) teacher-rated score was 9.5 (5.8) points; at 16 years, the mean (SD) NeuroTrax global score was 100.1 (14.8) points. Both in cluster-adjusted models and models also adjusted for baseline characteristics, the 6.5-year WASI full-scale IQ scores and 16-year NeuroTrax global cognitive scores were generally lower in offspring of mothers with higher late-pregnancy BMI quartiles (Figure). The highest BMI quartile was also associated with lower 11-year (but not 9-year) national examination scores. Associations were somewhat attenuated in the fully adjusted vs cluster-adjusted models. When associations existed, they tended to be linear, thus the remaining results are presented with BMI exposures modeled continuously.

**Figure.**
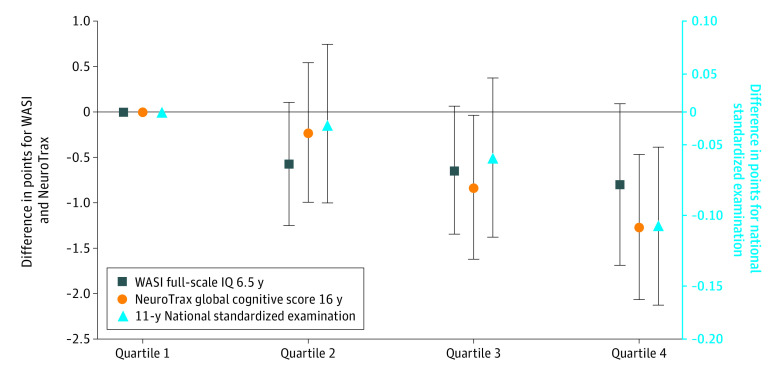
Adjusted Associations of Maternal Late-Pregnancy BMI in Quartiles With Selected Child Cognitive Outcomes Estimated associations (with error bars indicating 95% CIs) for the second, third, and fourth quartiles compared with the first BMI (lightest) quartile. Results from mixed effects linear regression models corrected for clustering within polyclinics and adjusted for geographic location, maternal age, height, and smoking; maternal and paternal education and occupation; number of older siblings; child sex; and treatment group.

In adjusted models with the exposure of continuous BMI, each 5-unit increase in maternal late-pregnancy BMI was associated with a 0.52-point decrement (95% CI, −0.87 to −0.17 points) in performance IQ and a 0.27-point decrement (95% CI, −0.61 to 0.07 points) in full-scale IQ. Higher maternal BMI was also associated with lower scores on multiple domains of the NeuroTrax battery, including attention, executive function, processing speed, memory, and verbal function, as well as the global (ie, total standardized) score (−0.67 points; 95% CI, −1.06 to −0.29 points) (Table 2). Continuous models also revealed more externalizing and overall behavioral problems (ie, total difficulties) on the teacher-reported SDQ, but not on the parent-reported SDQ (externalizing behavioral problems: 0.13 points; 95% CI, 0.02 to 0.24 points; overall behavioral problems: 0.14 points; 95% CI, −0.02 to 0.30 points) (Table 2).

**Table 2.** Associations of Late Pregnancy Maternal BMI (Continuous, per 5-unit Increment) With Child Cognitive and Behavioral Outcomes, Adjusted for Different Characteristics^a^

Cognitive and behavioral outcomes	No.	Mean (SD)	Effect estimate, (95% CI)			
			Fully adjusted^a^	Fully adjusted plus GDM, PIH^a^^,^^b^	Fully adjusted plus paternal BMI^a^^,^^b^^,^^c^	Fully adjusted plus child BMI^a^^,^^b^^,^^c^
WASI IQ at 6.5 y						
Full	10 497	106.6 (16.0)	−0.27 (−0.61 to 0.07)	−0.24 (−0.58 to 0.10)	−0.30 (−0.64 to 0.04)	−0.42 (−0.77 to −0.08)
Performance	10 503	107.1 (15.3)	−0.52 (−0.87 to −0.17)	−0.49 (−0.84 to −0.14)	−0.52 (−0.87 to −0.17)	−0.54 (−0.90 to −0.18)
Verbal	10 501	104.6 (17.1)	0.00 (−0.36 to 0.36)	0.03 (−0.34 to 0.39)	−0.04 (−0.41 to 0.32)	−0.24 (−0.61 to 0.13)
SDQ parent report at 6.5 y^d^						
Total difficulties	10 483	11.5 (5.0)	0.05 (−0.08 to 0.18)	0.04 (−0.10 to 0.17)	0.07 (−0.06 to 0.21)	0.03 (−0.10 to 0.17)
Internalizing	10 484	5.2 (3.0)	0.03 (−0.05 to 0.11)	0.02 (−0.06 to 0.10)	0.03 (−0.05 to 0.11)	0.03 (−0.06 to 0.11)
Externalizing	10 485	6.3 (3.2)	0.03 (−0.06 to 0.11)	0.02 (−0.07 to 0.10)	0.04 (−0.05 to 0.12)	0.01 (−0.08 to 0.10)
Prosocial	10 488	8.3 (1.6)	0.03 (−0.01 to 0.07)	0.03 (−0.01 to 0.08)	0.03 (−0.02 to 0.07)	0.02 (−0.02 to 0.07)
SDQ teacher report at 6.5 y^d^						
Total difficulties	9236	9.5 (5.8)	0.14 (−0.02 to 0.30)	0.13 (−0.03 to 0.29)	0.17 (0.01 to 0.33)	0.15 (−0.01 to 0.32)
Internalizing	9237	4.3 (3.0)	0.01 (−0.08 to 0.10)	0.01 (−0.07 to 0.10)	0.02 (−0.07 to 0.11)	0.04 (−0.05 to 0.13)
Externalizing	9238	5.2 (4.0)	0.13 (0.02 to 0.24)	0.12 (0.01 to 0.23)	0.15 (0.04 to 0.26)	0.12 (0.00 to 0.23)
Prosocial	9236	7.5 (2.2)	−0.02 (−0.08 to 0.04)	−0.02 (−0.08 to 0.05)	−0.03 (−0.09 to 0.03)	−0.02 (−0.09 to 0.04)
Neurotrax at 16 y						
Global standardized score	10 304	100.1 (14.8)	−0.67 (−1.06 to −0.29)	−0.67 (−1.06 to −0.28)	−0.72 (−1.11 to −0.33)	−0.68 (−1.07 to −0.28)
Attention	10 310	100.1 (14.8)	−0.74 (−1.13 to −0.35)	−0.73 (−1.12 to −0.33)	−0.75 (−1.14 to −0.35)	−0.79 (−1.20 to −0.38)
Executive function	10 302	100.2 (14.8)	−0.50 (−0.89 to −0.11)	−0.47 (−0.87 to −0.08)	−0.57 (−0.96 to −0.17)	−0.50 (−0.90 to −0.09)
Processing speed	9547	99.9 (14.9)	−0.57 (−0.98 to −0.15)	−0.56 (−0.98 to −0.14)	−0.55 (−0.97 to −0.13)	−0.60 (−1.02 to −0.17)
Memory	10 304	100.0 (15.0)	−0.42 (−0.82 to −0.02)	−0.40 (−0.81 to 0.00)	−0.45 (−0.86 to −0.05)	−0.35 (−0.77 to 0.06)
Motor skills	10 026	100.3 (14.7)	−0.06 (−0.45 to 0.33)	−0.06 (−0.45 to 0.33)	−0.13 (−0.52 to 0.26)	−0.16 (−0.56 to 0.25)
Verbal function	10 293	99.9 (15.0)	−0.62 (−1.02 to −0.23)	−0.63 (−1.03 to −0.23)	−0.64 (−1.04 to −0.24)	−0.61 (−1.02 to −0.21)
Visual/spatial	10 290	100.0 (15.0)	−0.21 (−0.61 to 0.19)	−0.19 (−0.59 to 0.21)	−0.24 (−0.65 to 0.16)	−0.16 (−0.58 to 0.25)
National examination scores^d^						
9-y examination scores	3345	6.7 (1.1)	−0.03 (−0.08 to 0.02)	−0.03 (−0.08 to 0.02)	−0.04 (−0.08 to 0.01)	−0.03 (−0.08 to 0.02)
11-y examination scores	5906	8.0 (1.1)	−0.05 (−0.09 to −0.02)	−0.05 (−0.09 to −0.02)	−0.06 (−0.09 to −0.02)	−0.05 (−0.08 to −0.01)

Abbreviations: BMI, body mass index (calculated as weight in kilograms divided by height in meters squared); GDM, gestational diabetes; IQ, intelligence quotient; PIH, pregnancy-induced hypertension; SDQ, Strengths and Difficulties Questionnaire; WASI, Wechsler Abbreviated Scales of Intelligence.

^a^ Results from mixed-effects linear regression models corrected for clustering within polyclinics. All models adjusted for geographic location; maternal age, height, and smoking; maternal and paternal education and occupation; number of older siblings; child sex; and treatment group.

^b^ Multiple imputation was used for missing data on GDM, preeclampsia, paternal BMI, and child BMI. All models have the same sample size across each outcome.

^c^ Paternal BMI by maternal reported height and weight at 6.5 years postpartum; child BMI by research measured child weight and height at 6.5 years.

^d^ Ranges: SDQ total difficulties 0-40; internalizing and externalizing behaviors 0-20; prosocial behaviors 0-10; national examination scores 2.5-10.

Higher maternal late-pregnancy BMI was associated with slightly lower scores on the 11-year national school examination scores (−0.05 points; 95% CI, −0.09 to −0.02 points) and weaker with the 9-year school examination results (Table 2), although more children participated in the 11-year examinations compared with the 9-year examinations (5906 vs 3345). The randomized breastfeeding intervention was not associated with school examination scores at 9 years (0.05 points; 95% CI, −0.22 to 0.32 points) or 11 years (0.09 points; 95% CI, −0.11 to 0.30 points) in intention-to-treat analyses.

Additional adjustment for the pregnancy complications of gestational diabetes and preeclampsia, for paternal BMI reported at 6.5 years, or for child BMI minimally attenuated associations of maternal late-pregnancy BMI with child outcomes (Table 2). Results were similar when we used prenatal maternal height obtained from obstetric records rather than research-measured height to calculate BMI. Results examining associations of maternal BMI at different time points were also consistent with our primary analyses, as expected given their strong intercorrelations (Table 3). We observed no consistent differences in magnitude of associations by sex (eTable 3 in the Supplement). Contrary to the consistent inverse findings with maternal BMI before, during, and after the PROBIT pregnancy, paternal BMI (n = 9546, reported by the mother at the 6.5-year study visit) was positively associated with child cognitive and behavioral outcomes (eTable 4 in the Supplement).

**Table 3.** Adjusted Associations of Maternal BMI (Continuous, per 5 kg/m^2^) at Different Time Points With Child Cognitive and Behavioral Outcomes^a^

Cognitive and behavioral outcomes	Estimate (95% CI)		
	First trimester, from prenatal record (n = 2346)	6.5 y, self-reported (n = 10 455)	11.5 y, research measure (n = 6398)
WASI IQ at 6.5 y			
Full	−0.34 (−1.03 to 0.35)	−0.31 (−0.59 to −0.03)	−0.08 (−0.35 to 0.19)
Performance	−0.88 (−1.63 to −1.13)	−0.34 (−0.63 to −0.05)	−0.06 (−0.34 to 0.22)
Verbal	0.18 (−0.52 to 0.87)	−0.24 (−0.54 to 0.06)	−0.08 (−0.37 to 0.20)
SDQ parent report at 6.5 y^b^			
Total difficulties	0.01 (−0.27 to 0.28)	0.12 (0.01 to 0.23)	0.09 (−0.02 to 0.19)
Internalizing	−0.03 (−0.19 to 0.14)	0.10 (0.03 to 0.16)	0.08 (0.01 to 0.14)
Externalizing	0.03 (−0.14 to 0.21)	0.02 (−0.05 to 0.09)	0.01 (−0.06 to 0.08)
Prosocial	0.10 (0.01 to 0.18)	0.04 (0.01 to 0.08)	0.04 (0.01 to 0.08)
SDQ teacher report at 6.5 y^b^			
Total difficulties	0.28 (−0.06 to 0.61)	0.04 (−0.10 to 0.17)	0.09 (−0.03 to 0.22)
Internalizing	0.10 (−0.09 to 0.28)	−0.01 (−0.08 to 0.06)	0.04 (−0.03 to 0.11)
Externalizing	0.18 (−0.05 to 0.41)	0.05 (−0.04 to 0.14)	0.05 (−0.03 to 0.14)
Prosocial	−0.10 (−0.23 to 0.03)	0.03 (−0.03 to 0.08)	−0.03 (−0.08 to 0.02)
Neurotrax at 16 y			
Standardized score	−0.16 (−1.88 to 0.23)	−0.61 (−0.94 to −0.28)	−0.56 (−0.87 to −0.25)
Attention	−0.43 (−1.27 to 0.42)	−0.51 (−0.85 to −0.18)	−0.47 (−0.79 to −0.16)
Executive function	−0.59 (−1.43 to 0.26)	−0.34 (−0.68 to −0.02)	−0.42 (−0.73 to −0.11)
Processing speed	−0.77 (−1.64 to 0.10)	−0.33 (−0.68 to 0.02)	−0.21 (−0.55 to 0.12)
Memory	−0.59 (−1.43 to 0.25)	−0.50 (−0.74 to −0.06)	−0.28 (−0.60 to 0.04)
Motor skills	−0.37 (−1.20 to 0.46)	−0.21 (−0.54 to 0.12)	−0.41 (−0.71 to −0.09)
Verbal function	−1.29 (−2.12 to −0.45)	−0.69 (−1.03 to −0.36)	−0.62 (−0.94 to −0.31)
Visual/spatial	−0.76 (−1.59 to 0.07)	−0.23 (−0.57 to 0.11)	−0.16 (−0.49 to 0.16)
National examination scores^b^			
9-y examination scores	−0.05 (−0.15 to 0.05)	−0.05 (−0.09 to −0.01)	−0.02 (−0.06 to 0.02)
11-y examination scores	−0.11 (−0.18 to −0.03)	−0.03 (−0.06 to 0.00003)	−0.03 (−0.06 to −0.004)

Abbreviations: BMI, body mass index; IQ, intelligence quotient; SDQ, Strengths and Difficulties Questionnaire; WASI, Wechsler Abbreviated Scales of Intelligence.

^a^ Results from mixed effects linear regression models corrected for clustering within polyclinics and adjusted for geographic location maternal age, height, and smoking; maternal and paternal education and occupation; number of older siblings; child sex; and treatment group.

^b^ Ranges: SDQ total difficulties 0-40; internalizing and externalizing behaviors 0-20; prosocial behaviors 0-10; national examination scores 2.5-10.

## Discussion

In this cohort study of longitudinal data with measured maternal BMI and multiple research measures of cognition across childhood and adolescence, children born to mothers with higher BMI had a slightly poorer performance on several cognitive and behavioral outcomes. Associations were robust to adjustment for measured baseline sociodemographic factors, pregnancy complications, and paternal BMI, and we observed no evidence for mediation by child BMI.

Our findings are consistent with systematic reviews on this topic.^9,22,23,24^ For example, a 2018 meta-analysis by Sanchez et al^9^ found that, compared with children born to mothers with normal weight before their pregnancy, children of mothers with prepregnancy obesity were 58% more likely to display cognitive or intellectual delay (OR, 1.58; 95% CI, 1.39-1.79), and 42% more likely to have an emotional or behavioral problems (OR, 1.42; 95% CI, 1.26-1.59). However, our results are not directly comparable to many studies that examined maternal weight before pregnancy, which was not measured in our cohort. Because our primary maternal weight measure was in late pregnancy, we cannot disentangle the distinct contributions of prepregnancy weight and gestational weight gain. Nevertheless, we consider our measure of clinically recorded weight to be a substantial strength of our analysis. In most prior studies, prepregnancy weight was recalled, and thus, susceptible to misclassification and social desirability biases.

Nevertheless, effect sizes were small in magnitude. For example, we have previously reported that individuals randomized to the PROBIT breastfeeding promotion intervention experienced improvements of 7.5 (95% CI, 0.8 to 14.3) points for verbal IQ, 2.9 (95% CI, −3.3 to 9.1) points for performance IQ, and 5.9 (95% CI, −1.0 to 12.8) points for full-scale IQ at 6.5 years,^14^ as well as 1.4 (95% CI, 0.3 to 2.5) points for verbal function and 1.2 (95% CI, 0.01 to 2.4) points for memory at 16 years.^20^ In this analysis, each 5-unit increase of maternal late-pregnancy BMI was associated with differences in scores that were 0.5 points or less.

Residual confounding by unmeasured sociodemographic or lifestyle characteristics remains a concern in all observational studies. Most prior studies were conducted in settings with high obesity prevalence and a strongly inverse relationship between sociodemographic characteristics and maternal obesity. In the PROBIT cohort, higher maternal late-pregnancy BMI did not track very strongly with lower sociodemographic status; thus, this analysis adds cross-context comparison.^25^ We also attempted to triangulate our results by comparing associations with maternal vs paternal weight. One would expect the unmeasured confounding factors to be similar for both mothers and fathers and therefore result in associations in the same direction. Although a stronger association with maternal vs paternal BMI might support a causal effect of the intrauterine environment on fetal brain development, it is difficult to postulate a confounder that would change the direction of association with paternal and maternal obesity. In contrast to other studies that found weaker negative associations between paternal BMI and child cognition and behavior, we observed that higher paternal BMI was positively associated with these outcomes.

If the association of offspring and behavior with maternal BMI is at least in part causal, multiple and complex mechanisms may be at play.^5^ Obesity has consistently been associated with systemic inflammation and altered circulating hormones, including corticosteroids, leptin, and insulin, and with risk of thyroid dysfunction, all of which may adversely affect the developing brain.^26,27,28^ Maternal obesity increases risks of pregnancy complications such as gestational diabetes, preeclampsia, preterm birth, and birth trauma, which have been shown to be associated with offspring neurodevelopment. In our analysis, adjustment for gestational diabetes and preeclampsia did not seem to be associated. Babies with low birth weight, preterm birth, or low Apgar scores were excluded from PROBIT, and thus these birth complications cannot explain the associations we observed. Breastfeeding is not likely to be a factor because it did not vary by late-pregnancy BMI, and we adjusted for randomized study arm.

### Strengths and Limitations

Our study has many strengths, including a large sample size, clinically recorded prenatal maternal weights, research measures of child cognition and behavior, and prolonged follow-up with low rates of attrition. Nevertheless, this study had limitations. We do not have a measure of preconception weight and, therefore, cannot disentangle the specific role, if any, of gestational weight gain. Generalizability may also be limited, given that all participants were Belarusian and rates of obesity were low; however, we also consider it a strength that the study was set in a region of the world not included in previous studies examining associations of maternal BMI with child developmental outcomes and may be less prone to confounding by socioeconomic or other factors. We excluded preterm and low birth weight infants, who may have poorer neurobehavioral outcomes, which may limit generalizability.

## Conclusions

The most clinically relevant implication of this analysis suggests that children born to women with obesity should be observed closely for neurodevelopmental problems and referred as appropriate for early intervention or other supportive services. Our results also have implications for future research into mechanisms by which environmental exposures, including maternal prenatal weight and nutritional status, can impact fetal brain development. Reversing the obesity epidemic for women of childbearing age, as for other segments of the population, will likely not be achieved by individual efforts, but will require governmental leadership, regulation, and investment.^29^ Given the strong associations of childhood intelligence and behavior with lifetime earning potential,^30,31^ our results support such initiatives.

## Notes

Supplement. **eTable 1.** Characteristics of PROBIT Participants Included vs Excluded From the Present Analysis **eTable 2.** Correlations of Maternal, Paternal, and Child BMI Measures at Different Timepoints **eTable 3.** Adjusted Associations of Late Pregnancy Maternal BMI (Continuous, Per 5 kg/m^2^) With Child Cognitive and Behavioral Outcomes, by Sex **eTable 4.** Associations of Paternal BMI (Continuous, Per 5 kg/m^2^), Assessed at 6.5 Years Postpartum by Maternal Report of Paternal Height and Weight With Child Cognitive and Behavioral Outcomes **eFigure.** Maternal and Paternal Occupation and Employment at Enrollment According to Quartiles of Maternal or Paternal BMI Assessed at Different Timepoints
