# Supplementary material for: Analysis of Maternal Prenatal Weight and Offspring Cognition and Behavior: Results From the Promotion of Breastfeeding Intervention Trial (PROBIT) Cohort
Source: JAMA Netw Open. 2021 Aug 19;4(8):e2121429. doi: 10.1001/jamanetworkopen.2021.21429 (PMC8377565; doi:10.1001/jamanetworkopen.2021.21429)
Supplement: Supplement. — eTable 1. Characteristics of PROBIT Participants Included vs Excluded From the Present Analysis eTable 2. Correlations of Maternal, Paternal, and Child BMI Measures at Different Timepoints eTable 3. Adjusted Associations of Late Pregnancy Maternal BMI (Continuous, Per 5 kg/m2) With Child Cognitive and Behavioral Outcomes, by Sex eTable 4. Associations of Paternal BMI (Continuous, Per 5 kg/m2), Assessed at 6.5 Years Postpartum by Maternal Report of Paternal Height and Weight With Child Cognitive and Behavioral Outcomes eFigure. Maternal and Paternal Occupation and Employment at Enrollment According to Quartiles of Maternal or Paternal BMI Assessed at Different Timepoints [file jamanetwopen-e2121429-s001.pdf]

Supplemental Online Content

Oken E, Thompson JW, Rifas-Shiman SL, et al. Analysis of Maternal Prenatal Weight and Offspring Cognition and Behavior: Results From the Promotion of Breastfeeding Intervention Trial (PROBIT) Cohort. *JAMA Netw Open.* 2021;4(8):e2121429. doi:10.1001/jamanetworkopen.2021.21429

eTable 1. Characteristics of PROBIT Participants Included vs Excluded From the Present Analysis
eTable 2. Correlations of Maternal, Paternal, and Child BMI Measures at Different Timepoints
eTable 3. Adjusted Associations of Late Pregnancy Maternal BMI (Continuous, Per 5 kg/m^2) With Child Cognitive and Behavioral Outcomes, by Sex
eTable 4. Associations of Paternal BMI (Continuous, Per 5 kg/m^2), Assessed at 6.5 Years Postpartum by Maternal Report of Paternal Height and Weight, With Child Cognitive and Behavioral Outcomes
eFigure. Maternal and Paternal Occupation and Employment at Enrollment According to Quartiles of Maternal or Paternal BMI Assessed at Different Timepoints

This supplemental material has been provided by the authors to give readers additional information about their work.

© 2021 Oken E et al. *JAMA Network Open.*

eTable 1. Characteristics of PROBIT Participants Included vs Excluded From the Present Analysis

	Included (N=11,276)	Excluded (N=5,770)
**Maternal characteristics**	**Mean (SD) or N (%)**	
Age at delivery, years		
<20	1471 (13.1%)	908 (15.7%)
20-34	9355 (83.0%)	4590 (79.6%)
>=35	450 (4.0%)	271 (4.7%)
Education		
Completed university	1506 (13.4%)	810 (14.0%)
Incomplete university	5827 (51.7%)	2743 (47.5%)
Completed Secondary	3570 (31.7%)	1927 (33.4%)
Incomplete secondary	373 (3.3%)	290 (5.0%)
Occupation		
Nonmanual	4923 (43.7%)	2316 (40.1%)
Manual	3817 (33.9%)	1817 (31.5%)
Student	321 (2.9%)	283 (4.9%)
Unemployed	2215 (19.6%)	1354 (23.5%)
Late pregnancy BMI, kg/m^2	27.2 (3.8)	26.9 (3.8)
**Paternal characteristics**		
Education		
Completed university	1381 (12.3%)	911 (15.8%)
Incomplete university	283 (2.5%)	172 (3.0%)
Completed Secondary	9029 (80.1%)	4285 (74.3%)
Incomplete secondary	224 (2.0%)	176 (3.1%)
Missing	359 (3.2%)	226 (3.9%)
Occupation		
Nonmanual	3161 (28.0%)	1694 (29.4%)
Manual	6098 (54.1%)	2866 (49.7%)
Student	138 (1.2%)	98 (1.7%)
Unemployed	1431 (12.7%)	799 (13.8%)
Unknown	448 (4.0%)	313 (5.4%)
**Participant Characteristics**		
Female	5421 (48.1%)	2787 (48.3%)
Birthweight (g)	3444 (416)	3425 (425)
Location of residence		
East, urban	3646 (32.3%)	1971 (34.2%)
East, rural	1446 (12.8%)	1260 (21.8%)
West, urban	2892 (25.7%)	1488 (25.8%)
West, rural	3292 (29.2%)	1051 (18.2%)

© 2021 Oken E et al. *JAMA Network Open.*

eTable 2. Correlations of Maternal, Paternal, and Child BMI Measures at Different Timepoints[a]

	Mother 1st trimester N=2,346	Mother late pregnancy N=11,276	Mother 6.5 years N=10,455	Mother 11.5 years N=6,398	Father 6.5 years N=9,546	Child 6.5 years N=10,526	Child 11.5 years N=10,549	Child 16 years N=10,375
	Pearson correlation coefficient (r)							
Mother 1st trimester	1	0.90	0.75	0.74	0.17	0.24	0.26	0.28
Mother 3rd trimester	0.90	1	0.68	0.70	0.13	0.22	0.23	0.24
Mother 6.5 years	0.75	0.68	1	0.76	0.20	0.18	0.22	0.23
Mother 11.5 years	0.74	0.70	0.76	1	0.13	0.18	0.25	0.24
Father 6.5 years	0.17	0.13	0.20	0.13	1	0.18	0.20	0.18
Child 6.5 years	0.24	0.22	0.18	0.18	0.18	1	0.68	0.55
Child 11.5 years	0.26	0.23	0.22	0.25	0.20	0.68	1	0.70
Child 16 years	0.28	0.24	0.23	0.24	0.18	0.55	0.70	1

[a] Limited to 11,276 with maternal late pregnancy BMI and any child cognitive/behavioral outcome

© 2021 Oken E et al. *JAMA Network Open.*

eTable 3. Adjusted Associations of Late Pregnancy Maternal BMI (Continuous, Per 5 Kg/M^2) With Child Cognitive and Behavioral Outcomes, by Sex[a]

Cognitive and behavioral outcomes	Boys N=5855	Girls N=5421	Boys, Fully adjusted*	Girls, Fully adjusted*	Interaction P value for sex
**WASI at 6.5 years**	Mean (SD)		Effect estimate (95% confidence interval)		
IQ full	106.5 (16.4)	106.6 (15.6)	-0.25 (-0.73, 0.23)	-0.31 (-0.79, 0.16)	0.71
IQ performance	107.4 (15.6)	106.8 (14.9)	-0.53 (-1.03, -0.03)	-0.54 (-1.03, -0.05)	0.61
IQ verbal	104.3 (17.4)	104.9 (16.7)	0.02 (-0.49, 0.53)	-0.02 (-0.53, 0.49)	0.74
**SDQ parent report at 6.5 years**					
Total	11.9 (5.0)	11.1 (4.9)	0.14 (-0.04, 0.33)	-0.03 (-0.22, 0.15)	0.87
Internalizing	5.1 (3.0)	5.3 (3.0)	0.03 (-0.08, 0.14)	0.03 (-0.09, 0.14)	0.25
Externalizing	6.7 (3.3)	5.8 (3.1)	0.12 (-0.01, 0.23)	-0.06 (-0.18, 0.06)	0.18
Prosocial	8.1 (1.7)	8.5 (1.6)	-0.01 (-0.07, 0.05)	0.07 (0.01, 0.13)	0.06
**SDQ teacher report at 6.5 years**					
SDQ total	10.6 (6.0)	8.3 (5.3)	0.30 (0.07, 0.53)	-0.04 (-0.26, 0.18)	0.26
Internalizing	4.4 (3.1)	4.2 (3.0)	0.02 (-0.10, 0.15)	-0.01 (-0.13, 0.12)	0.82
Externalizing	6.2 (4.2)	4.1 (3.5)	0.28 (0.12, 0.44)	-0.03 (-0.39, 0.61)	0.07
Prosocial	7.0 (2.3)	8.0 (2.0)	-0.07 (-0.16, 0.02)	0.04 (-0.04, 0.12)	0.15
**Neurotrax at 16 years**					
Standardized score	99.6 (15.4)	100.6 (14.2)	-0.69 (-1.25, -0.14)	-0.62 (-1.15, -0.09)	0.43
Attention	99.5 (15.5)	100.7 (14.1)	-0.74 (-1.31, -0.17)	-0.72 (-1.26, -0.18)	0.57
Executive function	100.8 (15.0)	99.6 (14.5)	-0.63 (-1.18, -0.08)	-0.34 (-0.89, 0.22)	0.19
Processing speed	99.0 (15.6)	100.7 (14.0)	-0.71 (-1.31, -0.11)	-0.40 (-0.96, 0.16)	0.19
Memory	98.4 (15.9)	101.6 (13.7)	-0.22 (-0.81, 0.37)	-0.64 (-1.17, -0.10)	0.47
Motor skills	103.1 (13.8)	97.2 (15.0)	-0.23 (-0.75, 0.28)	0.16 (-0.43, 0.75)	0.27
Verbal function	97.9 (15.6)	102.0 (14.0)	-0.66 (-1.24, -0.08)	-0.55 (-1.09, -0.01)	0.71
Visual/spatial	100.7 (15.3)	99.3 (14.5)	-0.13 (-0.69, 0.44)	-0.26 (-0.82, 0.30)	0.77
**National examination scores**					
19-year scores	6.4 (1.1)	7.0 (1.1)	-0.02 (-0.08, 0.04)	-0.06 (-0.13, 0.02)	0.53
11-year scores	7.8 (1.1)	8.3 (1.0)	-0.09 (-0.15, -0.04)	-0.02 (-0.06, 0.03)	0.01

[a] Results from mixed effects linear regression models corrected for clustering within polyclinics and adjusted for geographic location, maternal age, height, and smoking; maternal and paternal education and occupation; number of older siblings; and treatment group

BMI = body mass index; IQ = intelligence quotient; SDQ = Strengths and Difficulties Questionnaire; WASI = Wechsler Abbreviated Scales of Intelligence

© 2021 Oken E et al. *JAMA Network Open.*

eTable 4. Associations of Paternal BMI (Continuous, Per 5 kg/m^2), Assessed at 6.5 Years Postpartum by Maternal Report of Paternal Height and Weight, With Child Cognitive and Behavioral Outcomes

Cognitive and behavioral outcomes	Unadjusted	Adjusted*	Adjusted + late pregnancy maternal BMI
**WASI at 6.5 years**	Effect estimate (95% Confidence Interval)		
IQ Full	0.64 (0.24, 1.03)	0.16 (-0.22, 0.54)	0.21 (-0.18, 0.60)
IQ Performance	0.24 (-0.16, 0.63)	-0.15 (-0.54, 0.23)	-0.06 (-0.47, 0.34)
IQ Verbal	0.88 (0.46, 1.29)	0.42 (0.02, 0.82)	0.42 (0.00, 0.83)
**SDQ parent report at 6.5 years**			
SDQ total	-0.23 (-0.37,-0.09)	-0.17 (-0.31,-0.02)	-0.17 (-0.32,-0.02)
Internalizing	-0.10 (-0.18,-0.01)	-0.08 (-0.16, 0.01)	-0.07 (-0.16, 0.02)
Externalizing	-0.13 (-0.22,-0.04)	-0.09 (-0.18, 0.00)	-0.10 (-0.19, 0.00)
Prosocial	0.02 (-0.02, 0.07)	0.01 (-0.04, 0.06)	0.01 (-0.04, 0.06)
**SDQ teacher report at 6.5 years**			
SDQ total	-0.37 (-0.54,-0.19)	-0.29 (-0.46,-0.11)	-0.31 (-0.49,-0.13)
Internalizing	-0.13 (-0.22,-0.04)	-0.11 (-0.21,-0.02)	-0.11 (-0.21,-0.02)
Externalizing	-0.24 (-0.36,-0.11)	-0.18 (-0.29,-0.06)	-0.20 (-0.32,-0.07)
Prosocial	0.12 (0.05, 0.19)	0.09 (0.02, 0.16)	0.09 (0.02, 0.16)
**Neurotrax at 16 years**			
Standardized score	0.66 (0.21, 1.10)	0.35 (-0.08, 0.79)	0.45 (0.00, 0.90)
Attention	0.16 (-0.29, 0.60)	-0.07 (-0.52, 0.38)	0.06 (-0.40, 0.52)
Executive function	0.89 (0.45, 1.34)	0.68 (0.24, 1.12)	0.75 (0.29, 1.20)
Processing speed	-0.09 (-0.56, 0.37)	-0.33 (-0.80, 0.14)	-0.21 (-0.69, 0.28)
Memory	0.43 (-0.02, 0.88)	0.27 (-0.18, 0.72)	0.34 (-0.13, 0.81)
Motor skills	1.01 (0.56, 1.46)	0.79 (0.35, 1.23)	0.73 (0.28, 1.19)
Verbal function	0.26 (-0.18, 0.71)	0.06 (-0.39, 0.51)	0.15 (-0.32, 0.61)
Visual/spatial	0.55 (0.10, 1.00)	0.32 (-0.13, 0.77)	0.37 (-0.10, 0.84)
**National examination scores**			
9-year exam scores	0.09 (0.03, 0.14)	0.06 (0.01, 0.12)	0.07 (0.02, 0.13)
11-year exam scores	0.06 (0.02, 0.11)	0.04 (0.00, 0.08)	0.05 (0.01, 0.09)

* Results from mixed effects linear regression models corrected for clustering within polyclinics and adjusted for geographic location, maternal age, height, and smoking; maternal and paternal education and occupation; number of older siblings; child sex; and treatment group

© 2021 Oken E et al. *JAMA Network Open.*

eFigure. Maternal and Paternal Occupation and Employment at Enrollment According to Quartiles of Maternal or Paternal BMI Assessed at Different Timepoints

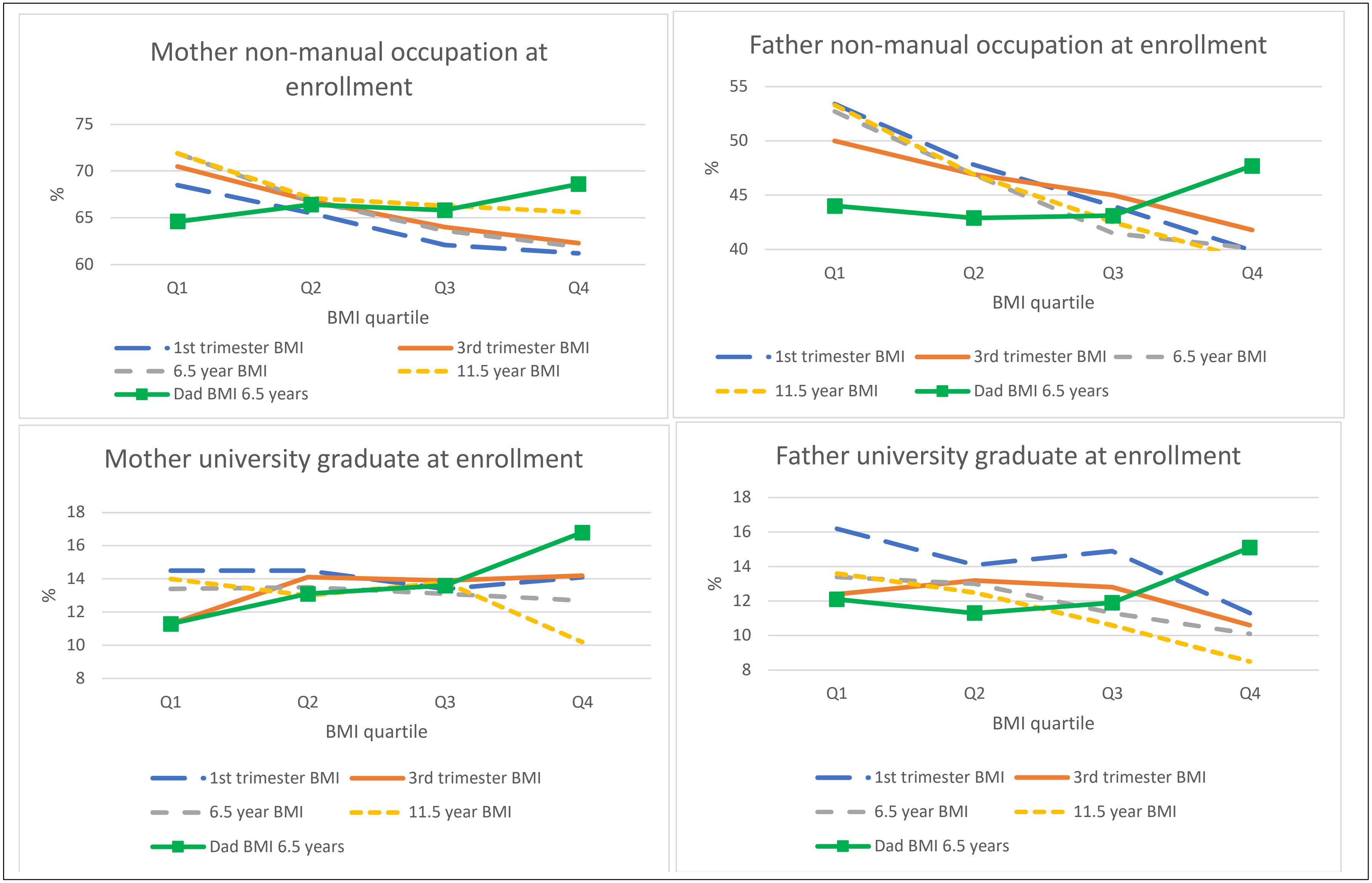

© 2021 Oken E et al. *JAMA Network Open.*
